# Vascular endothelial cells as signaling niches for epithelial stem cells in the skin

**DOI:** 10.3389/fcell.2026.1781544

**Published:** 2026-03-02

**Authors:** Tudorita Tumbar, Torsa Ganguly, Cailin E. McMahon, Mohammad A. Tavallaei

**Affiliations:** Department of Molecular Biology and Genetics, Cornell University, Ithaca, NY, United States

**Keywords:** cancer, homeostasis, perivascular niche, skin epithelial stem cells, stress, vascular endothelial niche

## Abstract

In many non-skin tissues, vascular endothelial cells (VECs) are increasingly recognized as integral components of stem cell niches engaged in bidirectional molecular crosstalk, regulating stem cell behavior through angiocrine signaling. In the skin, however, the nature and physiological relevance of VEC cues influencing epithelial stem cells remain poorly defined. While many cell types, including fibroblast, immune cells, nerves, and adipocytes are known to crosstalk to the epithelial stem cells in skin contributing to their niche, recent studies began to implicate VECs as a putative niche component. Indeed, skin epithelial stem cells can actively influence local vasculature via stage- and spatially restricted secretion of vascular remodeling factors. Conversely, genetic perturbations that enhance secretion of quiescence-inducing signals from the blood vessel VECs alter hair follicle stem cell proliferation and disrupt tissue homeostasis. Although these findings demonstrate that VECs can in principle modulate epithelial stem cell states, the specific signals and physiological contexts where VECs instruct skin stem cells remain largely unknown. Beyond homeostasis, VEC–stem cell interactions may in theory contribute to skin responses to environmental and pathological stresses, including ultraviolet irradiation, psoriasis, and cancer. Here we aim to raise awareness that, as observed in many non-skin tissues and tumors, skin VECs may likewise function not only as delivery conduits but also as putative signaling niches that shape epithelial stem cell states across diverse contexts. This review highlights an underexplored layer of vascular–epithelial crosstalk with potential relevance for skin homeostasis and disease, revealing a need for deeper mechanistic investigation in this research area.

## Introduction

Adult stem cells (SCs), such as epithelial skin SCs, are essential for tissue homeostasis and injury repair, relying on instructive cues from their specialized microenvironment, the SC niche. The skin niche is dynamically changing during aging (Chaudhary et al., 2025) and tumorigenesis (Pham et al., 2025). Extensive work has demonstrated that epithelial SC behavior in skin is a result of integrated signals from multiple niche components, including extracellular matrix, fibroblasts, immune cells, adipocytes and neural inputs (Li and Tumbar, 2021; Zhang and Chen, 2024). Although an “usual suspect” of many non-skin SC niches, vascular endothelial cells (VECs) have been relatively understudied in the skin, until very recently. The emerging interactions of epithelial skin SCs and VECs and the potential implications for various contexts, including homeostasis, stress, and cancer will be the focus of this review.

Two distinct classes of VECs form the lymphatic and blood vessels, presenting distinct cellular subsets with characteristic gene expression profiles and physiological functions (Ramasamy et al., 2015; Shimizu and Kubota, 2025). Many adult non-skin SC niches (e.g., hematopoietic, muscle, intestine, and neural) have recognized signaling contributions from VECs, particularly those of blood vessels but also lymphatics (Deng et al., 2023; Karakatsani et al., 2023; Shimizu and Kubota, 2025; Yang et al., 2025; Palikuqi et al., 2022). Traditionally viewed as conduits for oxygen, nutrients, and waste removal, VECs can regulate SC behavior through secretion of paracrine factors—collectively termed angiocrine signals (Rafii et al., 2016). These VEC-derived signals influence nearby SC activity during homeostasis, organogenesis and regeneration (Rafii et al., 2016; Ribatti et al., 2021), as well as in tumorigenesis and other pathological conditions (Butler et al., 2010). Some blood VEC derived signals in non-skin tissues promote nearby SC quiescence (Deng et al., 2023; Karakatsani et al., 2023; Shimizu and Kubota, 2025; Yang et al., 2025), counterbalancing the canonical blood vessel role in tissue growth.

While many adult SCs remain largely quiescent during homeostasis, others—such as intestinal and epidermal SCs—are highly proliferative and depend on distinctive niche-derived cues for regulation (Trentesaux et al., 2020). How VECs adapt to the divergent demands of these niches remains largely unresolved. Emerging evidence suggests that VECs function as molecular rheostats, integrating local signals from surrounding cells and extracellular matrix and adjusting their secretome accordingly (Ramasamy et al., 2015). Although VECs share a core endothelial lineage program, they also display tissue- and microenvironment-specific transcriptional signatures, implying tailored SC niche functions (Gomez-Salinero et al., 2025).

The skin harbors both quiescent hair follicle stem cells (HFSCs) in the bulge (Li and Tumbar, 2021) and more proliferative SCs in the basal layer of the inter-follicular epidermis (IFE) (Ishikawa et al., 2025). These two epithelial SC populations share molecular and functional similarities, but except during injury, they remain faithfully confined to their own niche (Sun et al., 2023). Both HFSC and IFE-SC pools display additional heterogeneity, with specific subsets displaying distinct characteristics that would prompt differential interactions with the SC niche (Blanpain and Fuchs, 2009; Ghuwalewala et al., 2022). For example, the hair germ stem cells are primed for activation and in direct contact with a mesenchymal signaling center, the dermal papilla (DP), whereas the bulge stem cells are more primitive and removed from the DP (Li and Tumbar, 2021; Zhang and Chen, 2024). Despite advances in other non-skin tissues, the defining features of skin VEC niches and their specific crosstalk with distinct subsets of epithelial SCs in physiological contexts relevant to skin biology remain poorly understood, especially for the IFE.

Here, we review emerging evidence of specific contexts where VECs may function as signaling niches for adult epithelial SCs, emphasizing distinct roles of blood versus lymphatic VECs. In fact, HFSCs are now well known to signal to VECs and to many other niche components, organizing their own skin microenvironment (Li and Tumbar, 2021). Although aspects of skin niche aging are complex and should be discussed in depth elsewhere, targeting the SC micro-environment is a proposed general therapeutic strategy in tissue aging (Farahzadi et al., 2023).

Excluding wound healing, which is covered elsewhere (Hur, 2024; Jiang and Perez-Moreno, 2024), we offer a perspective on the putative roles of a VEC – tissue epithelial stem cell signaling axis in several conditions: homeostasis, immunological and UV-induced stress, and cancer. Understanding the VEC-SC crosstalk in these conditions might prove one day to have physiological relevance for future therapies.

## Coordinated dynamics and crosstalk of VECs and hair follicle stem cells

**FIGURE 1:**
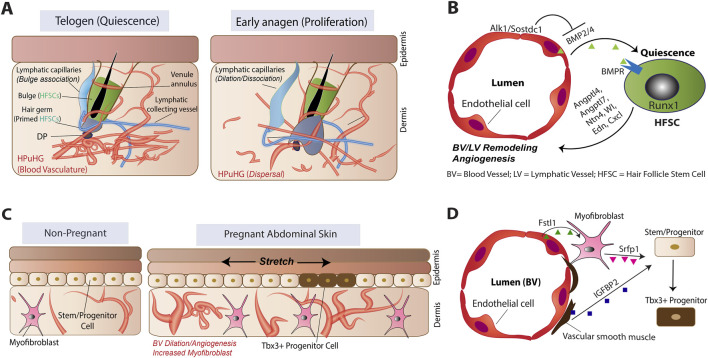
Schematic of vascular endothelial cell organization and epithelial stem/progenitor cell cross-talking during homeostasis. **(A,B)** Processes associated with hair follicle stem cell (HFSC) crosstalk in hair cycle. HPuHG, horizontal plexus underneath the hair germ. **(C,D)** Processes associated with interfollicular epidermis cross-talking during pregnancy.

Adult HFSCs spend most of their lifespan quiescent and are in active cross-communication with multiple essential niche components, including fibroblast, immune cells and nerves (Li and Tumbar, 2021; Zhang and Chen, 2024). VEC have only recently been recognized as possible niche components for the HFSCs, which reside adjacent to a stable venous structure, the venule annulus (Li and Tumbar, 2021; Xiao et al., 2013). The venule annulus remains structurally stable across the hair cycle—telogen, anagen, and catagen (Xiao et al., 2013) —while surrounding skin vasculature remodels dynamically in coordination with HFSC activation (Li and Tumbar, 2021; Mecklenburg et al., 2000; Yano et al., 2001) (Figure 1A). Based on other systems, stable perivascular association near veins may establish hypoxic microenvironments that promote SC quiescence (Deng et al., 2023; Karakatsani et al., 2023; Shimizu and Kubota, 2025; Yang et al., 2025), though this model is pending experimental evidence in the HFSCs case.

In human and mouse skin, blood vessels contact multiple hair follicle compartments, including the bulge SCs, the hair germ, the matrix progenitors, and the dermal papilla (Li and Tumbar, 2021). These interactions change over the hair cycle, particularly during anagen, when HF stem and progenitor cells proliferate to generate a new hair shaft, and at catagen, when the follicle regresses and HFSCs return to quiescence (Li and Tumbar, 2021). Early studies reported increased angiogenesis during anagen, and disrupting this process resulted in stunted hair follicles (Mecklenburg et al., 2000; Yano et al., 2001). Our BrdU pulse–chase analyses revealed that baseline angiogenesis persisted even during telogen, with at least one-third of VECs dividing during the first mouse hair cycle (Chovatiya et al., 2023).

We reported that most VEC expansion at anagen contributes to blood vessel arterial and capillary network growth, reflecting increased oxygen and nutrient demands and the metabolic reprogramming of VEC transcriptional programs during hair cycle (Chovatiya et al., 2023). At the same time lymphatic VEC numbers decline at anagen (Chovatiya et al., 2023), possibly reflecting shifts in fluid drainage activity (Jiang and Perez-Moreno, 2024; Gur-Cohen et al., 2019; Pena-Jimenez et al., 2019). The exact cellular source of new VECs during hair-cycle angiogenesis remains unresolved. A population of vascular endothelial progenitors with low VE-cadherin expression, described in wounds and tumors (Donovan et al., 2019; Patel et al., 2017), resembled to some extent a perineurial mesenchymal population we identified in skin (Chovatiya et al., 2023). However, lineage tracing showed that this population forms tubular structures that protect nerve bundles during skin homeostasis but did not contribute to homeostatic skin vasculature dynamics. Blood vessel VEC expansion during anagen is counterbalanced by apoptotic pruning at catagen, when the skin vasculature reorganizes into a horizontal plexus beneath the hair germ found at the dermis–hypodermis junction during telogen (Li et al., 2019; Li and Tumbar, 2021; Mecklenburg et al., 2000; Yano et al., 2001). This plexus disperses and reorients vertically at early anagen around the expanding HF bulb, a process likely dependent on Alk1-regulated VEC migration (Li et al., 2023; Rochon et al., 2016) (Figure 1A).

The dynamic structure and physiological functions of the skin lymphatic vasculature have been comprehensively reviewed elsewhere (Jiang and Perez-Moreno, 2024; Skobe and Detmar, 2000). Here, we briefly summarize its recently elucidated interactions with the HFSCs. Lymphatic capillaries reside near HFSCs along one side of the bulge during telogen but retract and open-up at anagen onset (Gur-Cohen et al., 2019; Pena-Jimenez et al., 2019). Ablation of lymphatic VECs impairs HFSC activation in both the hair germ and bulge, likely due to disrupted fluid drainage.

Lymphatic VECs may signal to HFSCs—for example, by secreting a BMP antagonist (Sostdc1) which would promote HFSC proliferation (Yoon and Detmar, 2022). Our lymphatic vessel–specific deletion of Alk1 upregulates BMP4 near HFSCs without immediate quiescence induction, possibly due to concomitant acceleration of lymphatic remodeling that prematurely adopts anagen morphology (Li et al., 2023). These findings raise the possibility that lymphatic drainage–mediated cues promoting HFSC activation at anagen may override potential inhibitory signals originating from lymphatic endothelium. Alternatively, heterogeneity of responses to lymphatic signals may occur in bulge vs. hair germ SCs due to distinctive distance from the lymphatic capillaries. Notably, transgenic mice lacking peripheral lymphatics throughout development display an apparently normal hair coat (Makinen et al., 2001), suggesting that compensatory mechanisms can sustain hair follicle function even in the absence of lymphatics.

BMP signaling is a key pathway enforcing HFSC quiescence. Loss of BMP receptor I in HFSCs leads to uncontrolled proliferation, whereas BMP ligands block proliferation of cultured keratinocytes (Botchkarev and Sharov, 2004; Genander et al., 2014; Lee and Tumbar, 2012; Plikus et al., 2008; Lee et al., 2021). VECs produce BMP ligands in several tissues, including neural and pancreatic systems (Mathieu et al., 2008; Jun et al., 2025; Saito et al., 2012), controlling neural SC quiescence among other functions. In skin, multiple sources—including dermal papilla, inner bulge epithelial cells, adipocytes, and a rare venous VEC subset—secrete BMP4 (Li et al., 2023; Li and Tumbar, 2021). Our endothelial-specific deletion of *Bmp2/4* in skin mildly impacts HFSC quiescence, while *Alk1* deletion induces upregulation of BMP ligand expressions in VECs. This promotes reprogramming of the HFSCs transcriptome in line with elevated BMP signaling, inducing quiescence and prolonged telogen (Li et al., 2023). Combined *Alk1/Bmp4* deletion in VECs partially restores HFSC activity, demonstrating the essential role of BMP signals originating from vasculature in *Alk1*-induced HFSC quiescence. *Alk1* loss in VECs affects proliferation in telogen HFs, but not in anagen, suggesting stage- and HF cell-type specific effects (Li et al., 2023; Li and Tumbar, 2021). This is important, as loss of *Alk1* can impair blood vessel remodeling and induce arteriovenous malformation (AVM) formation, which may affect vasculature physiological function (Roman and Hinck, 2017; Tual-Chalot et al., 2014). Finally, *Alk1* deletion in lymphatic VEC alone does not block HFSC activation, pointing to blood vessels as the source of quiescence inducing signals in this context (Li et al., 2023; Li and Tumbar, 2021) (Figure 1B).

Conversely, HFSCs, like other tissue SCs, actively signal to VECs, influencing their behavior and spatial organization during homeostasis (Li and Tumbar, 2021). First, angiogenic signals from the epithelium stimulate VEC proliferation during anagen, thereby enhancing nutrient and energy delivery to HF matrix cells (Mecklenburg et al., 2000). Second, hair cycle stage-specific levels of angiopoietins 4 and 7 in HFSCs, together with additional vascular remodeling factors such as netrin-4, modulate the organization and positioning of lymphatic capillaries across the hair cycle (Gur-Cohen et al., 2019). In addition, secretion of Wnt ligands from HFSCs is essential for lymphatic remodeling during the hair cycle (Pena-Jimenez et al., 2019). Finally, our work on loss or overexpression of RUNX1 in HFSCs disrupted blood vessel VEC remodeling and affected HFSC activation (Hoi et al., 2010; Li et al., 2019; Osorio et al., 2008). RUNX1 overexpression in the HFSCs alters their expression of angiogenic and vascular remodeling secreted factors, including *Ntn4*, *Sema3e*, *Edn1*, and *Figf* (Lee et al., 2014). This highlights a dual role of RUNX1 and its target genes to promote HFSC proliferation through both cell intrinsic and microenvironmental cues related to vasculature organization (Figure 1B).

In summary, HFSCs actively drive remodeling of their perivascular niche, consistent with their role as a central signaling hub coordinating skin homeostasis (Li and Tumbar, 2021). In addition, blood vessel VECs can act as signaling niches for HFSCs in specific contexts (Figures 1A,B).

It remains to be determined whether additional signals regulate HFSCs beyond the contexts described here, and how heterogeneous HFSC subsets may differentially interpret VEC-derived cues. Interestingly, the broad aging niche – including ECM, fibroblast, nerves, immune cells etc - has a dominant effect over skin epithelial stem cell intrinsic activity, as clearly shown by Ge et al. (2020). Moreover, VEC senescence was recently proposed to directly contribute to dermal skin aging (Wicaksono et al., 2025; Wu et al., 2010). This could, in principle, affect HFSC behavior in the long-term, an aspect of aging skin biology that would be interesting to investigate more in the future.

## VECs and interfollicular epidermis (IFE) interactions

The IFE is a stratified epithelium continuously renewed from basal proliferative cells, forming a protective body barrier (Gadre et al., 2024). Single-cell analyses and lineage tracing experiments revealed heterogeneity in the basal layer with distinct molecular profiles and self-renewal capacities, including distinct stem and progenitor populations, long- and short-term transit-amplifying cells, and differentiating basal cells (Aragona et al., 2017; Ghuwalewala et al., 2024; Ghuwalewala et al., 2022; Mascre et al., 2012; Sanchez-Danes et al., 2016; Cockburn et al., 2022). Lack of definitive markers for basal layer subpopulations has hindered niche characterization and the precise localization of IFE stem cells is currently unknown. Nevertheless, compelling evidence suggests that extracellular matrix composition, basement membrane interactions, and mechanical cues robustly support IFE SC function (Aragona et al., 2020; Biggs et al., 2020; Blanpain and Fuchs, 2009; Choi et al., 2015).

Although the IFE itself is avascular, basal cells receive oxygen and nutrients from the superficial papillary plexus (SPP), a dense capillary network in the upper dermis (Rimal et al., 2024). Unlike HF vasculature, the SPP remains static during homeostasis, at least in the hairless paw skin of mice (Kam et al., 2023). While evidence for direct VEC to IFE stem/progenitor signaling is scarce, perivascular cells such as pericytes and dermal mesenchymal cells are known to mediate vascular–epidermal communication (Zhuang et al., 2018). For example, pericytes enhance epidermal stem/progenitor cell self-renewal via basement membrane modification, particularly through LAMA5, which promotes keratinocyte proliferation in transplantation assays (Paquet-Fifield et al., 2009; Li et al., 2004).

Recently, Ichijo et al. reported that increased blood vessel density indirectly promotes the emergence of Tbx3^+^ transient progenitors in the abdominal epidermis during pregnancy and in paw epidermis (Ichijo et al., 2021; Ichijo et al., 2017) (Figure 1C). VECs upregulate FSTL1, promoting myofibroblast differentiation. The myofibroblast in turn secrete SFRP1 and this, together with IGFBP2 secretion by the vascular smooth muscle cells that line the walls of arteries, induce the generation of *Tbx3* ^+^ transient progenitor cells from the IFE SCs (Ichijo et al., 2021; Ichijo et al., 2017) (Figures 1C,D). Conversely, aging-associated vascular atrophy leads to dermal stiffening and Piezo1-mediated calcium influx, driving differentiation of IFE stem/progenitor cells (Ichijo et al., 2022). In addition, other age-related changes in the dermal microenvironment, including ECM remodeling, altered immune cell populations, and neural mis-localization, profoundly affect epithelial SC behavior (Ge et al., 2020), pointing to an urgent need for further investigation.

Thus, IFE stem/progenitor cell behavior is indirectly regulated by vascular cues transmitted through perivascular mesenchymal intermediates, enabling context-specific responses such as transient progenitor expansion during pregnancy or differentiation during aging.

## VEC–SC putative crosstalk in UV irradiation and psoriasis

**FIGURE 2:**
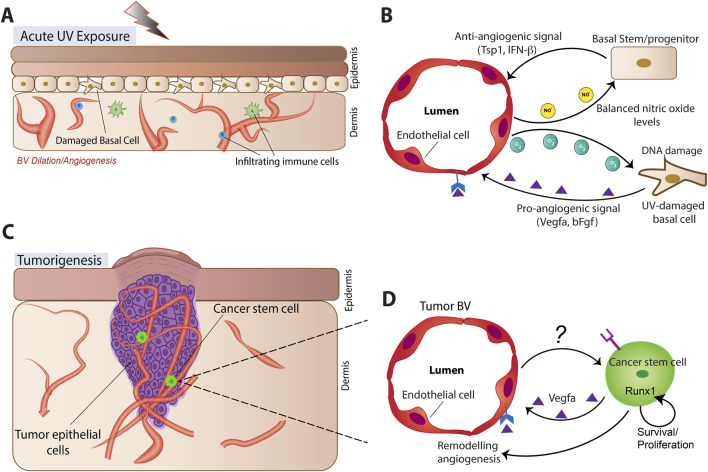
Schematic of vascular endothelial cell organization and epithelial stem/progenitor cell cross-talking during stress and pathological responses. **(A,B)** Processes associated with acute UV exposure. **(C,D)** Processes associated with tumorigenesis.

Skin suffers various stresses, including UV-irradiation and immune-driven inflammations in psoriasis. Skin vasculature remodels under such stressors, and it is possible that alternations in the VEC -SC signaling crosstalk may impact skin physiology, but this is currently poorly understood. Nevertheless, acute UV exposure triggers shifts in vascular signaling, contributing to the “sunburn” response (Kripke, 1994). UV-exposed keratinocytes upregulate pro-angiogenic factors such as VEGF-A and bFGF while reducing vascular quiescence signals such as TSP1 and IFN-β, promoting dilated vessels and proliferative VECs (Bielenberg et al., 1998; Hartono et al., 2022; Yano et al., 2005) (Figures 2A,B). Elevated VEGF-A also drives leaky lymphatic vessels (Kajiya et al., 2006). Given the lymphatic vessel permeability may affect HFSC regulation (Gur-Cohen et al., 2019; Pena-Jimenez et al., 2019), such vascular alterations could secondarily influence epithelial SC behavior in UV exposure. In addition, increased vascular permeability facilitates recruitment of elastase-containing leukocytes to UV-exposed skin, which degrades extracellular matrix proteins contributing to cutaneous photodamage effects (Yano et al., 2005). Notably, pharmacological blockade of VEGF signaling attenuates acute sunburn responses (Hartono et al., 2022), underscoring a possible contributing role of keratinocyte-driven angiogenesis in tissue stress responses (Figures 2A,B).

Beyond angiogenesis, UV exposure also induces VECs to secrete SCF1/c-kit, activating melanocytes and promoting pigmentation, supporting the concept that VECs may also function as signaling niches for melanocyte SCs (Kim et al., 2018). Endothelial nitric oxide (NO) signaling contributes to epidermal homeostasis, but UV-induced eNOS uncoupling generates reactive oxygen species, which are damaging to keratinocytes and activate cell cycle check points (Craig et al., 2010; Shackelford et al., 1999; Wu et al., 2010). While transient checkpoint activation is protective, prolonged activation can impair proliferation and drive premature exhaustion of the epidermal SC pool and compromise the SC niche (Panich et al., 2016). Given these observations, it is tempting to speculate that under specific stress conditions, VECs may be partially detrimental to the stem cell niche by altering the oxidative microenvironment.

Psoriasis provides a further example where pathological immune-driven inflammations may eventually result in abnormal vascular–epidermal coupling. Affecting nearly 4% of adults worldwide, psoriasis is characterized by extensive vascular remodeling with leaky vessel, increased leukocyte transmigration and inflammation (Skayem et al., 2025; Li et al., 2024). Activated CD4^+^ T cells induce keratinocytes to produce VEGF-A that increases angiogenesis, creating a feed-forward loop of vascularization and inflammation (Han et al., 2024; Huggenberger and Detmar, 2011). Reduced BMP4 in psoriatic skin and HFSC-intrinsic perturbations, such as cJun/JUNB depletion, can contribute to hyperplasia and inflammation (Di Costanzo et al., 2019; Gago-Lopez et al., 2019). Finally, epidermal basal stem/progenitor cells retain long-lasting inflammatory memory via chromatin accessibility, accelerating responses to subsequent insults (Larsen et al., 2021; Naik et al., 2017). While this has not been yet directly linked to VECs *per se*, it is possible in principle that abnormal vascularization upon inflammation may contribute to epidermal SC altered states and epigenetic memories. Thus, the possibility that alterations in the skin vasculature may contribute to chronic disease and could in principle shape SC behavior is an intriguing possibility that requires further investigation.

## VECs as putative signaling niches in skin tumorigenesis

Multiple niche components and pathways have been described to regulate (skin) cancer stem cells, and the tumor microenvironment in many ways can highjack the normal stem cells signaling pathways (Pham et al., 2025; Rafii et al., 2016). While the broad tumor microenvironment has been addressed elsewhere (Pham et al., 2025; Rafii et al., 2016), we focus here on discussing the theoretical possibility and the scarce evidence that VEC crosstalk with epithelial SCs may be important in skin cancers. Vascular remodeling and angiogenesis are central features of solid tumor growth, which require substantial metabolic resources supplied by blood. Indeed, most tumors must induce neovascularization to expand beyond ∼2 mm in diameter (Folkman, 1975). VEC heterogeneity across tissues suggests they can adopt region-specific functional states likely exploited by tumors (Gomez-Salinero et al., 2025; Butler et al., 2010). Seminal studies demonstrated that many carcinomas upregulate VEGF, a factor now recognized as master regulator of angiogenesis in both developmental and pathological contexts (Ferrara, 2002) (Figures 2C,D). Distinctive vascular patterns are used to diagnose and predict outcomes for different skin tumor types, including basal cell carcinoma (BCC), squamous cell carcinoma (SCC), and melanoma (Giacomel and Zalaudek, 2005; Lupu et al., 2019; Zalaudek et al., 2010), suggesting possible tumor-type specificity of VEC remodeling. Current evidence has demonstrated that cancer stem cells (CSC) may also utilize a perivascular niche for their survival (Figure 2C). In SCC, CSC secrete VEGF which stimulates angiogenesis in a paracrine manner thus creating a perivascular niche for CSC. In addition, VEGF also acts directly on CSCs through Nrp1 in an autocrine loop, thus stimulating cancer stemness and renewal (Beck et al., 2011) (Figures 2C,D).

Beyond tumor-derived angiogenic cues, oncogenes expressed in epithelial SCs can also exert non–cell-autonomous effects on vascular remodeling. As mentioned in the HFSC section, our work on RUNX1 demonstrates that its regulation of target genes play a role in cell-extrinsic maintenance of vascular organization nearby the SC niche during hair cycle (Lee et al., 2014; Li et al., 2019). In addition, RUNX1 target genes also promote proliferation and a growth-favorable metabolic state in adult HFSCs (Hoi et al., 2010; Jain et al., 2018; Lee et al., 2013; Lee et al., 2014; Osorio et al., 2008). Furthermore, clonally marked Runx1+ HFSCs can generate the entire epithelial portion of a tumor suggesting they represent a tumor-initiating CSC. Perhaps not coincidentally, RUNX1 is overexpressed in many epithelial tumors and is absolutely required for squamous cell tumorigenesis in skin (Scheitz et al., 2012) while its expression is dispensable in normal skin. Thus, tumors appear to exploit the pleiotropic effects of RUNX1 in promoting stem cell activation through both cell intrinsic and angiogenic cues and become addicted to it. These results highlight the possibility that oncogene activity may simultaneously act both cell-intrinsically in cancer (stem) cells and extrinsically by shaping VECs in the tumor microenvironment (Figures 2C,D).

Classical paradigms posit that increased vascularity promotes tumor aggressiveness, consistent with evidence that VEC-derived signals support tumor growth (Butler et al., 2010). However, emerging data challenge an exclusively pro-tumorigenic role for blood vessels. Under homeostatic conditions, epithelial SC populations residing in perivascular niches remain predominantly quiescent. If we draw a parallel between normal and pathological conditions, it may follow that perivascular niches could promote quiescence, maintaining CSCs in a constrained state. This has in fact been demonstrated in glioblastoma (Calabrese et al., 2007), which we discuss here briefly as it can inform research in skin cancer biology. Glioblastoma, an aggressive and nearly uniformly lethal brain tumor, is thought to arise from neural stem or progenitor cells (Alcantara Llaguno et al., 2019). Tumor heterogeneity drives therapeutic resistance, with subsets of CSCs capable of regenerating tumors upon transplantation (Vescovi et al., 2006). These CSCs share morphological and transcriptional features with undifferentiated neural stem and progenitor cells (Urban et al., 2019). In the adult brain, neural SCs maintain direct contact with VECs, where EPHRINB2- and JAGGED-1–mediated interactions preserve quiescence (Ottone et al., 2014; Urban et al., 2019). Similarly, glioblastoma CSCs cluster around CD34^+^ vasculature, though the molecular signals governing this interaction remain incompletely defined (Calabrese et al., 2007). One VEC-derived factor of particular interest is Semaphorin 3G (SEMA3G). Elevated SEMA3G expression correlates with improved survival in glioblastoma patients, and recent evidence shows that VEC-derived SEMA3G suppresses CSC proliferation by reducing stability of the survival factor c-MYC downstream of NRP2 signaling (Min et al., 2025). These findings broadly align with work from our group in hair cycle showing that VECs can exert context-dependent, quiescence-inducing effects on nearby HFSCs (Li et al., 2023; Li et al., 2019). These considerations, although preliminary, highlight the need to further consider skin vascular niches as potential modulators of CSC behavior (Figures 2C,D).

Interestingly, the vascular factor ALK1 is upregulated in several cancers and predicts poor outcomes in glioblastoma and colorectal cancer (Bavi et al., 2013; Elsers et al., 2021). Accordingly, multiple ALK1-targeting agents are currently in clinical trials, largely aimed at disrupting its roles in vascular remodeling and angiogenesis (Poei et al., 2024). Given that ALK1 loss in skin VECs induced HFSC quiescence via BMP signaling (Li et al., 2023; Li et al., 2019), an important avenue for future investigation is whether ALK1 inhibition might also normalize VEC-derived angiocrine signals in skin tumors. Such an effect could in theory restrain tumor progression not only by limiting nutrient supply, but also by maintaining CSCs in a more quiescent state potentially disfavoring tumor growth.

## Discussion

The skin’s barrier function imposes unique challenges for tissue homeostasis, requiring precise coordination between epithelial SCs and their microenvironment. A balanced interplay of multiple microenvironmental cues from various cell types ensures continuous SC renewal in the IFE while hair follicles cycle between proliferation and quiescence. Skin vasculature is only a recent candidate in the research field studying the complex milieu that comprise the skin stem cells niche, yet it is a compelling one. The skin vasculature is dynamic, actively remodeling during the hair cycle, undergoing angiogenesis and pruning, and shifting the balance of specific VEC subsets with may provide a rich substrate for differential interactions with spatially and molecularly heterogeneous subsets of the epithelial stem cells. Furthermore, perturbations in blood or lymphatic vessels organization clearly can disrupt SC behavior, yet by mechanisms largely unknown. Detailed mechanistic studies in both homeostatic and disease contexts are needed in future to elucidate these mechanisms.

Collectively, the evidence discussed in this review supports the notion that VECs can in principle serve as signaling niches for HFSCs ina context-dependent manner, with the best example provided by our VEC-specific targeting of the ALK1-BMP signaling axis in mice (Li et al., 2023). We speculate that in ALK1 deficiency, as occurs in human hereditary hemorrhagic telangiectasia (HHT), where arteriovenous malformations (AVMs) frequently arise in the skin (Roman and Hinck, 2017; Ruiz-Llorente et al., 2017), mutant VECs might upregulate BMP ligands, consistent with observations from our mouse genetic models. This upregulation may delay HFSC activation and anagen progression, processes normally coupled to extensive vascular remodeling (Li et al., 2019), potentially exacerbating AVM formation in skin. Thus, skin vasculature may harbor a HFSC-inhibitory “backup” mechanism through the ALK1–BMP signaling axis, limiting potentially deleterious consequences of hair cycle in the pathological settings of *Alk1* loss.

Outside of this pathological context, however, the specific pathways and physiological conditions under which VECs of both blood and lymphatic vessels may function as *bona fide* signaling niches for HFSCs remain undefined. In addition to UV-irradiation and psoriasis, skin tumors are especially good instances for studying possible consequences of dysregulated vascular–SC interactions. Basal cell carcinoma (BCC), squamous cell carcinoma (SCC), and melanoma collectively account for approximately 10,000 deaths annually, with up to 20% of adults developing skin cancer by age 70 (Stern, 2010). Increasing evidence suggests that epithelial SCs within the hair follicle and interfollicular epidermis (Kudelka et al., 2025; Youssef et al., 2017; White and Lowry, 2015) and in some contexts melanocyte SCs (Centeno et al., 2023), can serve as cells of origin for skin tumors. Tumorigenesis in these contexts may involve profound shifts in SC–niche dynamics, as shown for other niche components in skin and other tissues. Currently a speculative model, the possibility remains that essential aspects of the tumor pathology may be putatively linked to altered skin VEC signaling, to promote - unexpectedly – cancer SC quiescence, as demonstrated for example, in glioma cancer SCs or in other non-skin SC systems.

This review article points to current emerging evidence on the potential role of vascular niches in skin, providing rational and future directions for new areas of investigation in homeostasis, stress responses, and tumorigenesis. Such insights could prove useful not only for understanding fundamental skin stem cell biology, but also for developing vascular-targeted therapies in dermatological and oncological diseases.
